# Alexithymia Is a Key Mediator of the Relationship Between Magical Thinking and Empathy

**DOI:** 10.3389/fpsyt.2021.719961

**Published:** 2021-08-24

**Authors:** Clare M. Eddy, Peter C. Hansen

**Affiliations:** ^1^Birmingham and Solihull Mental Health NHS Foundation Trust and College of Medical and Dental Sciences, University of Birmingham, Birmingham, United Kingdom; ^2^Centre for Human Brain Health and School of Psychology, University of Birmingham, Birmingham, United Kingdom

**Keywords:** emotion, empathy, social cognition, magical thinking, thought action fusion, obsessive - compulsive disorder, alexithymia, personal distress

## Abstract

Thought action fusion (TAF), whereby internal thoughts are perceived to exert equivalent effects to external actions, is a form of magical thinking. Psychiatric disorders associated with TAF (e.g. schizophrenia; obsessive compulsive disorder) can feature atypical social cognition. We explored relationships between TAF and empathy in 273 healthy young adults. TAF was directly correlated with higher personal distress, but not perspective taking, fantasy or empathic concern. TAF moral (the belief that thinking about an action/behaviour is morally equivalent to actually performing that behaviour) was predicted by emotion contagion, alexithymia and need for closure. TAF likelihood (the belief that simply having a thought about an event makes that event more likely to occur) was predicted by personal distress, sense of agency and alexithymia. Both cognitive (TAF and negative sense of agency) and emotional (emotion contagion, alexithymia) factors contributed to personal distress. TAF, negative sense of agency and personal distress mediated the effect of emotion contagion on alexithymia. Our findings reveal complex relationships between emotional processes and TAF, shedding further light on the social cognitive profile of disorders associated with magical thinking. Furthermore, they emphasise the potential importance of alexithymia and emotion contagion as mediators or potential risk factors in the development of psychiatric symptoms linked to TAF, such as intrusive thoughts about harm to others.

## Introduction

Magical thinking refers to a belief in personal power to control or cause external events in the real world beyond culturally and rationally accepted laws of causality (1). In a clinical sense, magical thinking characterises people who believe that their thoughts, words or action could in some manner, cause a specific outcome in a way that defies the normal laws of cause and effect (2). More specifically, Thought Action Fusion (TAF) refers to “the tendency to assume incorrect causal relationships between one's own thoughts and external reality” (3). TAF is divided into two components. “Likelihood TAF” is the belief that simply having a thought about an event makes that event more likely to occur, whereas “Moral TAF” is the belief that thinking about an action or behaviour is morally equivalent to actually performing that behaviour (4).

It is thought that magical thinking is central to Obsessive Compulsive Disorder (OCD) (5), and numerous studies have reported a link between TAF specifically and OCD symptoms (6–9), although relationships are more likely to occur with likelihood TAF than moral TAF (4, 10, 11). Likelihood TAF (but not moral TAF) is thought to be related to schizotypal traits even after controlling for negative affect and OCD (11). Furthermore, TAF can be related to auditory hallucinations (12, 13) and is a risk factor for the development of psychosis (14).

There are a few reasons why one may expect magical thinking to be related to aspects of social cognition, including empathy. Firstly, in the case when magical thinking involves other people, social cognition and thought action fusion may be most clearly intertwined. Secondly, psychiatric disorders that feature magical thinking also tend to be associated with atypical social cognition. For example, OCD can be associated with increased personal distress when witnessing someone else experiencing a crisis (15, 16) and some studies report differences to controls in terms of mirroring others' emotions (17), emotion recognition (18–20) or theory of mind i.e., reasoning about others' thoughts and emotions (21, 22). The presence of social cognitive impairment is established in schizophrenia (23). Patients with paranoid schizophrenia report significantly greater negative emotion contagion in comparison to controls (24) and risk markers for the development of psychosis include a combination of odd beliefs/magical thinking and impaired social function manifesting as anhedonia/asociality and inappropriate affect (14). Other symptoms further imply a close relationship between TAF and social cognition. For example, delusions related to telepathy involve both magical thinking and social cognition, and TAF could also help to explain paranoia or persecutory delusions on the basis that internal negative thoughts are erroneously assumed to reflect external negative actions and intentions (25). Specifically, OCD has been associated with increased empathic concern (15) and such tendencies could be related to worry about harm occurring to others.

The current study aimed to explore relationships between TAF and empathic processes including perspective taking, empathic concern, and emotional contagion. Understanding more about the relationship between social cognition and magical thinking is important for a number of reasons. One is that we may start to understand what causes certain clinical symptoms to co-occur, and whether one factor is causative or a risk factor for developing the other. We may then be able to predict social cognition based on a measure of thought action fusion or vice versa, allowing us to better predict related symptoms or behaviours such as the social cognitive problems, disturbing intrusive thoughts or compulsions of individuals who demonstrate magical thinking. Furthermore, we may be able to identify potential mediating factors, which could be targets for therapeutic intervention. For example, helping individuals to learn how to control their emotional responses could help with compulsive behaviours resulting from magical thinking.

In addition to the Thought Action Fusion Scale (9), we included the Interpersonal Reactivity Index (26, 27), to assess both the cognitive aspects of empathy (perspective taking, fantasy) and the affective aspects (empathic concern, personal distress). We also included the Emotion Contagion Scale (28) to look at mirroring or emotional resonance with others. Furthermore, we included the Toronto Alexithymia Scale (29, 30), as alexithymia (i.e., difficulties with identifying and describing internal emotional states) may in turn influence social cognition (31) and can be seen in disorders associated with magical thinking [OCD (16, 32, 33); schizophrenia (34, 35)]. In order to explore which factors may mediate the relationship between TAF and empathy, we included a couple of other scales relevant to conditions such as OCD and schizophrenia that are likely to be related to magical thinking. The first was the Need for Closure Scale (36, 37) which assesses a form of cognitive bias around intolerance of uncertainty. Low tolerance of ambiguity is thought to be associated with magical thinking (38) and is pertinent to OCD [Obsessive Compulsive Cognitions Working Group (39, 40)], helping to explain repetitive actions (41). The second was the Sense of Agency Scale (42). Both OCD (43–45) and schizophrenia (46, 47) have been associated with disordered agency attribution, and agency attribution may prompt mental state attribution (48). We expected that high TAF would be associated with high need for closure and low sense of agency. We also hypothesised that there would be a direct relationship between TAF and empathy. More specifically, we expected TAF would predict some aspects of empathy, but that a relationship in the reverse direction may be less likely. This is because while magical thinking can have clear social or emotional connotations (49) (e.g., intrusive thoughts about harm coming to others), empathy occurs within the general population in the absence of magical thinking, and the latter is considered to be a rather unusual trait [prevalence of <2% in non-psychotic psychiatric disorders (50)]. In addition, we predicted a possible mediation of the relationship between TAF and empathy by emotion contagion. Being exposed to other's emotions can lead to mirroring of those emotions and emotion contagion (51). In the case that this contagion is of a negative emotion, this process may result in personal distress. A relationship between TAF and emotion contagion may be expected given that the concept of thoughts and actions “fusing” parallels the fusing of a mirrored motor act such as a facial expression, and its corresponding internal emotional state. Finally, we explored whether alexithymia could arise as a result of high emotion contagion and personal distress (i.e., excessive emotional mirroring), plus altered sense of agency (e.g., loss of self-other distinction), as previously hypothesised (25). This may be expected given that experiencing emotions through emotion contagion and the mirroring of external stimuli rather than in response to personally experienced events could lead to confusion when trying to interpret emotions originating from the self. Alexithymia could also mediate the relationship between TAF and empathy, given that a reduction in emotional awareness as seen in alexithymia could affect future emotional responses in the form of personal distress or emotion contagion (25).

## Materials and Methods

### Participants

We recruited 297 participants (253 females, 44 males; mean age = 19.19 years; SD = 1.21; median = 19; range = 18–29) currently studying at the University of Birmingham. Recruiting from this participant pool allowed us to sample larger numbers for our modelling, and the TAF and IRI were both developed in non-clinical samples (9, 26), although variations in scores on these measures can be seen when comparing clinical and non-clinical samples. The study was granted by University of Birmingham ethical review board and conducted in accordance with the World Medical Association Declaration of Helsinki. All participants gave written informed consent.

### Materials and Procedure

Participants provided demographical information and then completed paper questionnaires: Interpersonal Reactivity Index (IRI), Toronto Alexithymia Scale (TAS), Thought Action Fusion Scale (TAFS), Emotion Contagion Scale (ECS), Sense of Positive and Negative Agency Scale (SOAS) and Need for Closure Scale (NFCS).

#### Interpersonal Reactivity Index

The IRI (26, 27) contains 4 subscales each with 7 items. Perspective taking assesses the tendency to adopt other people's points of view, and empathic concern addresses feelings of warmth and consideration toward others. High scores for personal distress indicate more feelings of negative emotion when around other people in distress and the fantasy subscale measures the tendency to imagine and relate to characters in books and films. Participants respond on a 5 point Likert scale based on how well-each item describes them. Some items are reverse scored, and total score ranges from 0 to 112.

#### Toronto Alexithymia Scale

The TAS-20 assesses alexithymia and has good reliability and construct validity (29, 30). There are three subscales: difficulty identifying emotions (DIF e.g., “I have feelings that I can't quite identify”); difficulty describing emotions (DDF e.g., “It is difficult for me to find the right word for my feelings”) and externally oriented thinking (EOT e.g., “I prefer to just let things happen rather than to understand why they turned out that way”). Twenty items are rated on a 5 point Likert scale from “strongly agree” to “strongly disagree.” Some items are reverse scored. Scores can range from 20 to 100, with scores of 61+ being proposed to identify alexithymic individuals, and above 51 probable/borderline alexithymia.

#### Thought Action Fusion Scale

The TAFS (9) assesses two aspects of TAF: the likelihood that thinking about something will make it more likely to happen (TAF likelihood, 7 items e.g., “If I think of a relative/friend being in a car accident this increases the risk that he/she will have a car accident”); and that thinking about doing specific thing is morally equivalent to doing that same thing (TAF moral, 12 items e.g., “Having violent thoughts is almost as unacceptable to me as violent acts”). Items are rated using a scale from “disagree strongly” (0) to “agree strongly” (4). Scores can range from 0 to 76.

#### Emotion Contagion Scale

The ECS (28) measures susceptibility to other people's emotions. It contains 15 items such as “It irritates me to be around angry people.” Items pertain to happiness, anger, fear, love or sadness. Each item is rated “never” (1), “rarely” (2), “often” (3) or “always” (4), with possible score ranging from 15 to 60.

#### Sense of Positive and Negative Agency Scale

The SOAS is suggested by the authors (42) to measure beliefs about being agents in terms of experiencing control over one's body, thought and immediate environment. The scale assesses both the sense of negative (e.g., “Nothing I do is actually voluntary”) and positive (“the decision whether and when to act is within my hands”) agency. It contains 13 items rated from “strongly disagree” (1) to “strongly agree” (7). We used the two individual subscales (sense of agency: positive, SOAP: 6 items; sense of agency: negative, SOAN: 7 items) and scores could range from 13 to 91 (SOAP: up to 42; SOAN up to 49).

#### Need for Closure Scale

This scale (37) contains 47 items rated from “strongly disagree” (1) to “strongly agree” (6). Items cover order (e.g., “I hate to change my plans at the last minute”), predictability (e.g., “I prefer to socialise with familiar friends because I know what to expect from them”), decisiveness (“I tend to struggle with most decisions”), ambiguity (e.g., “I dislike unpredictable situations”) and closed mindedness (e.g., “I always see many possible solutions to problems I face”). Some items are reverse scored. A subset of 5 questions in the 47 is intended to identify liars. Total score can range from 42 to 252.

### Analysis

After checking for missing data, we excluded all individuals scoring above the recommended threshold on the lie detector subscale of the NFCS (to exclude potentially unreliable data), leaving data from 273 participants. We computed two-tailed Pearson correlations (SPSS version 26.0) to examine relationships among all measures, which were used to inform regressions. Path analyses models were then constructed to identify variables mediating the effect of TAF on empathy, and other variables on TAF subscales using AMOS for SPSS (version 26.0). We used the bootstrap method (500 samples, 95% bias corrected confidence intervals) to calculate indirect effects due to higher power and good type I error control (52–54). Model fit was assessed using chi-square test (χ2), comparative fit index (CFI), root mean-squared error of approximation (RMSEA) and standardised root mean squared residual (SRMR). A non-significant χ2, CFI of 0.9 or above, RMSEA of 0.08 or less, and SRMR of below 0.05 indicate good model fit (55–57).

## Results

### Descriptive Statistics and Correlational Analyses

Descriptive statistics for scale data are shown in Table 1. TAFS scores spanned a wide range with 60 participants scoring at least 30, the mean score found by Shafran et al. (9) in a sample with OCD. There was also a good range of TAS scores, with 35 participants (13%) scoring above the clinical cut-off for the TAS of 61 or more and a further 52 (19%) scoring at least 51, which is suggested as borderline alexithymic (58). Mean ECS score was 45 (range 25–58) which was high in comparison to a previous study reporting mean ECS scores of 33.8 in healthy controls and 38.6 in schizophrenia (24) but not higher than other studies involving a high proportion of healthy females (28). There was a mean score for personal distress of approx. 14 (range 1–28). Previous mean scores for personal distress in healthy controls have been both lower (10.45) (15) and higher (15.8) (24). Means for individuals with schizophrenia (24) or OCD (15) have been reported as 20.6 and 17.32, respectively. In the current sample 81 participants (30%) scored 17 or above. TAF total was correlated with IRI personal distress, TAS, ECS, SOAN and NFC scores (in addition to its subscales). In relation to the TAFS subscales, TAFS moral was correlated with IRI empathic concern and personal distress, TAS, SOAN, NFC and TAFS likelihood scores, while TAFS likelihood was also correlated with personal distress, alexithymia and SOAN (Table 2).

**Table 1:** Descriptive statistics for each scale.

**Measure**		**Mean (standard deviation)**	**Median (Range)**	**Scale min; max**	**McDonald's Omega**
IRI	PT	19.46 (4.74)	20 (5–28)	0–28	0.721
	EC	21.98 (4.31)	22 (8–28)	0–28	0.784
	FS	18.77 (5.30)	19 (5–28)	0–28	0.764
	PD	13.98 (4.77)	14 (1–28)	0–28	0.759
TAFS	Total	20.05 (11.93)	18 (0–55)	0–76	0.833
	Likelihood	4.29 (5.54)	2.0 (0–27)	0–28	0.913
	Moral	15.77 (9.23)	15 (0–41)	0–48	0.885
TAS	Total	45.84 (11.29)	45 (22–81)	20–100	0.832
ECS	Total	44.99 (6.04)	33 (25–58)	15–60	0.757
SOAP		30.21 (5.87)	31 (10–42)	0–42	0.712
SOAN		17.19 (6.07)	17 (7–43)	0–49	0.742
NFCS	Total	159.52 (17.48)	161 (101–210)	42–252	0.753

*IRI, Interpersonal Reactivity Index; PT, Perspective Taking subscale; EC, Empathic Concern subscale; ECS, Emotion Contagion Scale; FS, Fantasy Subscale; PD, Personal Distress subscale; TAF, Thought Action Fusion Scale; TAS, Toronto Alexithymia Scale (20 item); SOAP, Sense Of Agency Positive subscale; SOAN, Sense Of Agency Negative subscale; NFCS, Need For Closure Scale*.

**Table 2:** Correlations between all measures.

**Measure**	**PT**	**EC**	**FS**	**PD**	**TAF T**	**TAF L**	**TAF M**	**TAS**	**ECS**	**SOAP**	**SOAN**
IRI PT	X										
IRI EC	0.486, <	X									
IRI FS	0.185, 0.002	0.306, <	X								
IRI PD	−0.113, 0.063	0.101, 0.095	0.059, 0.330	X							
TAF T	0.045, 0.464	0.079, 0.195	0.022, 0.720	0.262, <	X						
TAF L	−0.032, 0.597	−0.039, 0.526	0.040, 0.515	0.241, <	0.663, <	X					
TAF M	0.077, 0.206	0.125, 0.040	0.004, 0.943	0.193, 0.001	0.893, <	0.257, <	X				
TAS	−0.334, <	−0.295, <	−0.110, 0.069	0.211, <	0.197, 0.001	0.209, <	0.129, 0.034	X			
ECS	0.335, <	0.547, <	0.269, <	0.225, <	0.235, <	0.082, 0.174	0.254, <	−0.240, <	X		
SOAP	0.126, 0.037	0.146, 0.016	−0.016, 0.798	−0.210, <	0.028, 0.645	−0.028, 0.639	0.053, 0.381	−0.317, <	0.232, <	X	
SOAN	−0.216, <	−0.236, <	0.024, 0.688	0.212, <	0.212, <	0.208, 0.001	0.149, 0.014	0.394, <	−0.044, 0.465	−0.345, <	X
NFC	−0.258, <	−0.091, 0.135	−0.067, 0.272	0.206, 0.001	0.199, 0.001	0.093, 0.124	0.201, 0.001	0.097, 109	0.025, 683	0.055, 0.364	0.066, 0.274

*KEY: “<” denotes p 0.0001. IRI, Interpersonal Reactivity Index; PT, Perspective Taking subscale; EC, Empathic Concern subscale; FS, Fantasy Subscale; PD, Personal Distress subscale; TAF T, Thought Action Fusion Scale Total; TAF L, Thought Action Fusion Scale Likelihood subscale; TAF M, Thought Action Fusion Scale Moral subscale; TAS, Toronto Alexithymia Scale (20 item); ECS, Emotion Contagion Scale; SOAP, Sense Of Agency Positive subscale; SOAN, Sense Of Agency Negative subscale; NFCS, Need For Closure Scale*.

### Regressions and Path Analysis With Empathy Measures as Dependent Variables

**Figure 1:**
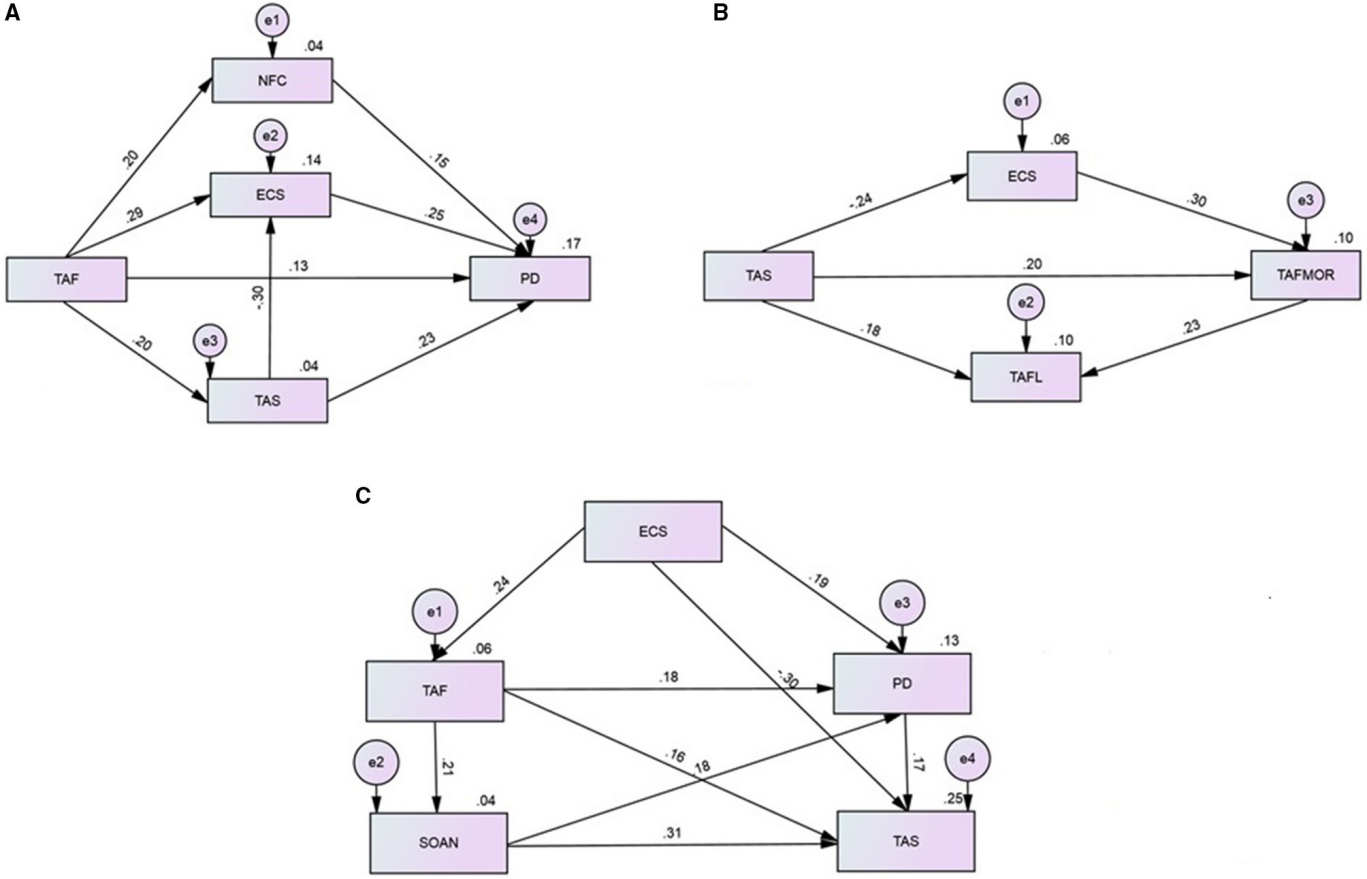
Mediation models showing the relationship between thought action fusion and empathy. **(A)** Mediation model showing how the effect of TAF on IRI PD subscale scores is mediated by NFC, ECS and TAS scores. **(B)** Mediation model showing the relationships between TAS, ECS and TAF subscale scores. **(C)** Mediation model showing the relationships between TAS, ECS, IRI PD, SOAN and TAF scores. Coefficients are shown along each path and R^2^ is given above each variable on the right hand side. All paths shown were statistically significant and error variance is shown as “e1” etc. PD, Personal Distress subscale score (of the Interpersonal Reactivity Index); ECS, emotion contagion scale total score; NFCS, need for closure scale total score; SOAN, sense of agency negative subscale score; TAF, thought action fusion scale total score; TAF L, thought action fusion scale likelihood subscale score; TAFM, thought action fusion scale moral subscale score; TAS, toronto alexithymia scale total score (20 item).

Only IRI empathic concern and personal distress were correlated with TAF. Therefore, regressions were conducted to determine how TAFS scores predicted empathic concern and personal distress, with models including other variables found to also correlate with empathic concern and personal distress, respectively, as IVs. With empathic concern as DV (IVs: IRI fantasy; TAFS moral; TAS; ECS), IRI fantasy, TAS and ECS scores significantly contributed but TAFS moral dropped out of the regression model. With personal distress as DV (IVs: TAFS total; TAS; ECS; NFCS) all IVs including TAFS total significantly contributed, therefore a mediation model was constructed (χ^2^(2) = 1.001, *p* = 0.606; CFI = 1.00; RMSEA = 0.000; SRMR = 0.0163) which explained 17% of the variance in personal distress. Emotion contagion, need for closure and alexithymia partially mediated the effect of TAF on personal distress (Figure 1A). Emotion contagion partially mediated the effect of alexithymia on personal distress, and alexithymia partially mediated the effect of TAF on emotion contagion.

### Regressions and Path Analysis With TAF as Dependent Variable

Regressions were then conducted using the TAFS subscales as DVs, to explore the potential for a bidirectional relationship between TAF and empathy (and for comparison to the hypothesised models with empathy measures as DV) including in the models other variables found to also correlate with the TAFS subscales, as IVs. When TAFS moral was DV (IVs: IRI empathic concern; IRI personal distress; TAFS likelihood; TAS; ECS; SOAN; NFC), TAFS likelihood, TAS, ECS and NFCS scores were significant predictors. With TAFS likelihood as DV (IVs: IRI personal distress; TAFS moral; TAS; SOAN) personal distress and TAFS moral scores were significant predictors. A mediation model was constructed including both TAFS subscales. The predictors NFCS and personal distress were removed to improve model fit. The final mediation model (χ^2^(1) = 1.535, *p* = 0.215; CFI = 0.992; RMSEA = 0.044; SRMR = 0.0209) which explained 10% of the variance in each TAF subscale, showed that emotion contagion partially mediated the effect of alexithymia on TAFS moral, and TAFS moral partially mediated the effect of alexithymia on TAFS likelihood (Figure 1B).

### Regressions and Path Analysis With Alexithymia as Dependent Variable

Finally, we tested whether alexithymia could arise as a result of high ECS, SOAN, and high personal distress, and how this related to TAF. The model (χ^2^(1) = 2.690, *p* = 0.101; CFI = 0.988; RMSEA = 0.079; SRMR = 0.0269) explained 25% of the variance in alexithymia, showing that personal distress partially mediated the effect of emotion contagion on alexithymia, TAFS total partially mediated the effect of emotion contagion on personal distress, negative sense of agency partially mediated the effect of TAFS on personal distress, SONA partially mediated the effect of TAFS on alexithymia, and personal distress mediated the effect of SOAN on alexithymia (Figure 1C).

## Discussion

TAF total scores were positively correlated with IRI personal distress, but were not correlated with the perspective taking or the fantasy subscales. IRI empathic concern was correlated with TAF moral but not TAF likelihood or TAF total scores. In addition, TAF was correlated with emotion contagion. Therefore, TAF in general appears to be related to the affective aspects of empathy but perhaps not the cognitive aspects, perhaps because TAF involves fusion between mental concepts and actions, and emotions are often expressed as observable actions. That is, magical thinking is considered to utilise a pre-symbolic mode of thought, given that it depends on the existence of an object rather than a mental representation (59), and emotional expressions constitute action objects in a way that abstract mental representations (e.g., beliefs) usually cannot.

The relationship between TAF and personal distress is in line with previous studies reporting elevated personal distress in conditions associated with magical thinking such as OCD (15, 16), schizophrenia (60–64) and Tourette syndrome (65). The selective association between empathic concern and TAF moral could suggest that this aspect of TAF is related more to the intention to be empathic toward others, whereas TAF likelihood is perhaps associated with more generalised emotional reactivity in response to others. We expected TAF may predict empathic response but that the converse may be less likely, given that empathy seems to occur frequently within the general population in the absence of magical thinking, but TAF sometimes has clear social or emotional connotations, such as concern about harm coming to others (49). Although we cannot confirm causality, this possibility is in accordance with our findings. There was evidence that empathy in the form of personal distress was predicted by TAF in general, whereas inclusion of personal distress did not make for a good model fit when predicting TAF subscale scores. However, emotion contagion was a predictor of likelihood TAF. The potential value of this characteristic in predicting intrusive thoughts about harm to others in OCD samples is worthy of further research.

TAF total scores were also positively associated with alexithymia. Previous studies have suggested that both magical thinking and alexithymia may occur in disorders such as schizophrenia, and psychosis risk appears to be related to a combination of magical thinking and social dysfunction (14). However, the current study may be the first study to report a specific relationship between TAF and alexithymia. This relationship may suggest that the fusion of thoughts and external actions leads to confusion in relation to the interpretation of one's own internal states (emotions; visceral responses). Relationships were also found between alexithymia and personal distress, in line with previous studies (66, 67).

TAF total scores were associated with negative (i.e., low) sense of agency and high need for closure. It makes sense for TAF to be associated with negative sense of agency, given potential confusion between thoughts and actions and the originator of a perceived effect. Indeed, disorders involving magical thinking are associated with erroneous agency attribution (43–47), perhaps helping to explain patients' impulsive behaviours. The perceived inflated sense of responsibility seen in OCD may also be linked to sense of agency (68). While need for closure was related to TAFS moral, it wasn't correlated with TAFS likelihood. This could be because the intention to be moral involves both understanding and taking greater personal responsibility for actions whereas TAF likelihood may be more generally linked to anxiety and harm avoidance.

Path analyses showed that emotion contagion, need for closure and alexithymia were partial mediators of the effect of TAF on personal distress. Therefore, both cognitive and emotional factors can influence whether thought action fusion leads to personal distress. In OCD, intrusive thoughts about harm occurring to others and personal distress around this may be influenced by tendencies toward need for closure and a predisposition to emotion contagion. Within this model, emotion contagion partially mediated the effect of alexithymia on personal distress, and alexithymia mediated the influence of TAF on emotion contagion. In sum, this suggests that personal distress is predicted by emotion contagion, which in turn can be predicted by a lack of clarity in relation to one's own emotions. The finding that TAF can directly predict personal distress implies that this IRI subscale picks up on automatic emotional reactions which occur more in individuals who have a tendency toward confusing thoughts and actions. Emotion contagion also partially mediated the effect of alexithymia on TAF moral specifically, further highlighting the potential importance of emotional resonance, in addition to internal emotional awareness, in the experience of judging thoughts about harm occurring to others. This in itself supports the proposal that certain social cognitive strategies or modes (e.g., mirroring others rather than abstract reasoning or mentalizing about others) may be more intrinsically linked to thought action fusion (25).

The interrelationships identified in the current study between emotion contagion, alexithymia, and personal distress also support the possibility that in at least some cases, alexithymia could develop in response to high personal distress (25). We found that TAF and sense of agency further mediate these relationships, with our model explaining a sizable proportion (25%) of the variance in alexithymia scores. Greater emotion contagion, combined with thought action fusion in terms of linking the observed action to a mental state may help us to understand and empathise with emotions as expressed by others. However, if this is combined with difficulties in determining agency for that mental state, we could start to feel confused about the origin or ownership of emotions in a way that could result in high personal distress (25). Personal distress in turn could lead to maladaptive social behaviours, in addition to contributing to the development of alexithymia. However, given that alexithymia was found to be a negative predictor of emotion contagion, this could mean that in turn, alexithymia can reduce personal distress through lowered attention to emotional state and emotional blunting. These possibilities encourage further research.

Limitations of the current study include lack of inclusion of additional measures of more abstract forms of social cognition (e.g., false belief tasks) which would have helped to fully evaluate the relationship between TAF and social cognition. In addition, our sample was predominantly female. Our methods allowed us to explore the strengths of relationships between variables but further experimental work is required to establish causality and the presence of similar relationships within clinical populations.

In conclusion, there are multiple and complex relationships between TAF, alexithymia, emotion contagion, and empathy in the form of personal distress. Sense of agency and need for closure are cognitive factors that further interact with these variables. Understanding more about the relationship between empathy and magical thinking can shed light on why certain clinical symptoms co-occur, predictive risk factors, and potential compensatory mechanisms or coping strategies. Furthermore, the identification of potential mediating variables can highlight targets or outcome measures for therapeutic intervention. Our findings suggest that there is likely to be a specific relationship between TAF and affective empathy, such that individuals who experience TAF may be likely to show a social cognitive profile influenced by high emotion contagion and personal distress. These latter characteristics may therefore underlie variability in performance across different social cognitive tasks in patient populations who demonstrate high levels of magical thinking. We have further shown that TAF may encourage the development of alexithymia and personal distress, but that difficulties with interpreting one's own emotional state may predict likelihood TAF. This makes sense when we consider that TAF involves fusion between internal mental states and external actions/events. In addition, alexithymia may both result from high emotion contagion and/or personal distress, and contribute to these empathic processes, suggesting that alexithymia may manifest as a mediator, or regulatory mechanism against uncontrolled emotional reactivity toward social stimuli. Taken together, our findings compel follow up of these observations in clinical populations, which could highlight a potential benefit of combined interventions targeting both problematic thought action fusion (e.g., intrusive thoughts in OCD) and emotional reactivity (emotion contagion, alexithymia) in cases featuring both magical thinking and emotional dysregulation.

## Data Availability Statement

The raw data supporting the conclusions of this article will be made available by the authors, without undue reservation.

## Ethics Statement

The studies involving human participants were reviewed and approved by University of Birmingham Research Ethics Committee. The patients/participants provided their written informed consent to participate in this study.

## Author Contributions

CE developed the study concept, performed the data analysis and interpretation, and drafted the paper. Testing and data collection were performed by PH. PH conducted statistical review and other critical revisions. All authors contributed to the article and approved the submitted version.

## Conflict of Interest

The authors declare that the research was conducted in the absence of any commercial or financial relationships that could be construed as a potential conflict of interest.

## Publisher's Note

All claims expressed in this article are solely those of the authors and do not necessarily represent those of their affiliated organizations, or those of the publisher, the editors and the reviewers. Any product that may be evaluated in this article, or claim that may be made by its manufacturer, is not guaranteed or endorsed by the publisher.
